# The incidence of COVID-19 infection following emergency use authorization of BBIBP-CORV inactivated vaccine in frontline workers in the United Arab Emirates

**DOI:** 10.1038/s41598-021-04244-1

**Published:** 2022-01-11

**Authors:** Nawal Al Kaabi, Abderrahim Oulhaj, Farida Ismail Al Hosani, Shamma Al Mazrouei, Omer Najim, Salah Eldin Hussein, Jehad Saleh Abdalla, Mohammed Saifuddin Fasihuddin, Afnan Abdellatif Hassan, Gehad Elghazali, Ahmed Al Rumaithi, Jumana Al Azazi, Stefan Weber, Rami Beiram, Khatija A. Parekh, Mohamud Sheek-Hussein, Yunkai Yang, Yang Xiaoming, Jenny Quliang, Islam Eltantawy, Sally Mahmoud, Ashish Koshy, Peng Xiao, Subhashini Ganesan, Wael Elamin, Walid Zaher

**Affiliations:** 1 Sheikh Khalifa Medical City SEHA, Abu Dhabi, UAE; 2 Department of Epidemiology and Public Health, College of Medicine and Health Sciences, Khalifa University, Abu Dhabi, UAE; 3 Institute of Public Health, College of Medicine and Health Sciences, United Arab Emirates University, Abu Dhabi, UAE; 4 Abu Dhabi Public Health Center – ADPHC, Abu Dhabi, UAE; 5 Department of Health, Abu Dhabi, UAE; 6 Sheikh Khalifa Medical City-Union 71/Purehealth, Abu Dhabi, UAE; 7 College of Medicine and Health Sciences, United Arab Emirates University, Abu Dhabi, UAE; 8 China National Biotec Group Company Limited, Beijing, China; 9 G42 Healthcare, Masdar City, Abu Dhabi, UAE; 10 IROS (Insights Research Organization & Solutions), Masdar City, Abu Dhabi, UAE

**Keywords:** Medical research, Epidemiology, Outcomes research

## Abstract

Based on the findings from the Phase III clinical trials of inactivated SARS COV-2 Vaccine, (BBIBP-CORV) emergency use authorization (EUA) was granted for the vaccine to frontline workers in the UAE. A prospective cohort study was conducted among frontline workers to estimate the incidence rate and risk of symptomatic COVID-19 infection 14 days after the second dose of inoculation with BBIBP-CORV inactivated vaccine. Those who received two doses of the BBIBP-CORV vaccine in the period from 14th of September 2020 (first dose) to 21st of December 2020 (second dose) were followed up for COVID-19 infections. 11,322 individuals who received the two-dose BBIBP-CORV vaccine were included and were followed up post the second dose plus fourteen days. The incidence rate of symptomatic infection was 0.08 per 1000-person days (95% CI 0.07, 0.10). The estimated absolute risk of developing symptomatic infection was 0.97% (95% CI 0.77%, 1.17%). The confirmed seroconversion rate was 92.8%. There were no serious adverse events reported and no individuals suffered from severe disease. Our findings show that vaccinated individuals are likely to remain protected against symptomatic infection or becoming PCR positive for SARS COV 2 following the second dose of the vaccination.

## Introduction

The coronavirus disease 2019 (COVID-19) pandemic caused by severe acute respiratory syndrome coronavirus 2 (SARS-CoV-2) has infected more than 251 million people, with deaths exceeding 5 million worldwide, according to the World Health Organization report on November 2021^1^. The development of safe and effective vaccines against SARS-CoV-2 remains a global priority among efforts to control the COVID-19 pandemic and reduce the number of deaths caused by this disease.

The Sinopharm product BBIBP-CORV is an inactivated vaccine and is currently approved by the WHO in Emergency Use Listing (EUL)^2^. Preliminary findings of randomized, double-blinded, placebo-controlled phase 1 and 2 clinical trials to assess two different β-propiolactone-inactivated whole virus vaccines show that the BBIBP-CORV, vaccine was safe and well tolerated. In these trials, individuals reported a low rate of vaccine-related adverse reactions and demonstrated neutralizing antibody levels similar with those achieved with other vaccine candidates^3–5^.

Supported by these encouraging findings, and preliminary data from phase 3 trial, the United Arab Emirates (UAE) Ministry of Health and Prevention (MOHAP) granted an Emergency use authorization (EUA) for the inactivated SARS COV-2 Vaccine, BBIBP-CORV to frontline workers during September 2020 as an early attempt to protect individuals at high risk of infection^6^.

The aim of this study is to estimate the incidence rate and risk of symptomatic and total COVID-19 infection 14 days after the second dose and to describe the antibody response (i.e. seroconversion) and the side effects experienced by the participants following vaccination.

## Materials and methods

### Study design and settings

A prospective cohort study was conducted among frontline individuals (including but not limited to health care workers, police staff and blue-collar labourer’s) at an increased risk of infection with SARS COV2, who received two doses of the BBIBP-CORV vaccine in the period between 14th of September 2020 (first dose) to 21st of December 2020 (last dose); and attended SEHA healthcare facilities in Abu Dhabi (UAE) for vaccination.

At Day 0, individuals had an initial clinical assessment, a baseline blood test for antibodies (Anti S total antibody) and received their first dose of vaccination intramuscularly with the BBIBP-CORV vaccine (4 μg). This was followed by a telephone assessment within 7 days. Between days 21–28 they received their second dose of vaccination (BBIBP-CORV vaccine −4 μg) following assessment by the nursing team. At day 35, the individuals were evaluated by a physician and the antibody test was repeated. (Anti S quantitative antibody). The detailed schedule of events is shown in Table 1.

**Table 1:** Schedule of follow up events.

visit number	1	2	3	4	5
Day	**0**	**7**	**21**	**28**	**35**
Visit window (+ -/- days)	0	+ 7	+ 7	+ 7	+ 14
Participant consent	✔				
Inclusion/exclusion criteria	✔		✔		
Demographics	✔				
Medical history	✔		✔		
Administer vaccination	✔		✔		
Vital signs	✔		✔		✔
Body weight	✔		✔		✔
Post vaccination follow up call		✔		✔	
Adverse event	✔	✔	✔	✔	✔
Laboratory testing					
Pregnancy testing	✔		✔		
Anti SARS COV 2 assay	✔				✔
Neutralization antibody					✔

### Participants and follow-up

Eligible individuals were healthy adults 18 years and older with no history of COVID-19 infection, satisfying the national criteria for frontline workers at high to moderate risk of exposure to COVID-19, and who received the COVID-19 vaccine and attended the SEHA health care facilities following the EUA. As per the national guidelines, vaccination was not recommended for individuals with (1) history of severe COVID 19 infection requiring oxygen during admission, (2) Lactating women with a baby less than 6 months old, (3) Pregnant women or planning to be pregnant within 3 months, (4) History of severe reaction associated with vaccine e.g., anaphylaxis, (5) Bleeding disorders, (6) Live attenuated vaccine within 30 days, (7) Killed vaccine within 14 days, (8) Recent history of Guillain Barre Syndrome GBS) or transverse myelitis in the past 12 months, or (9) Active cancer or in remission for less than 6 months.

### Vaccine candidate

The vaccine administered to individuals was BBIBP-CORV, an inactivated SARS-CoV-2 vaccine. It is prepared by purification and passage and further expansion of the BBIBP-CORV strain in Vero cells. Inactivation is performed with β-propionolactone and aluminium hydroxide adjuvant is added^4^. This vaccine is prepared as a ready to use vaccine and no dilution is required. It is the first COVID-19 vaccine to be provided with a vaccine vial monitor, which makes it easy for the health workers to evaluate the viability of the vaccine. The vaccine is recommended to be stored in original package in a refrigerator at + 2 to + 8 °C. The easy storage requirements make it highly suitable for low resource settings^7^.

### Side effects monitoring

Local and systemic adverse events were documented on every encounter with the participants during the vaccination and by telephone follow up within one week of receiving the first dose and two weeks after the second dose in the follow up clinic. Local adverse events reported included pain, induration, swelling, rash, flush, pruritus. Systemic adverse events included fever, diarrhea, constipation, dysphagia, anorexia, vomiting, nausea, muscle pain (non-vaccination sites), arthralgia, headache, cough, dyspnea, pruritus at non-vaccination sites (no skin damage), skin and mucosal abnormalities, acute allergic reactions, fatigue.

### Outcomes

The primary outcomes were:

- Symptomatic COVID-19 infection defined as the first occurrence of symptomatic Covid-19 infection with onset at least 14 days after the second injection; confirmed by the PCR detection of SARS COV2 virus.
- COVID-19 infection defined as the first occurrence of a Covid-19 infection with onset at least 14 days after the second injection; confirmed by the PCR detection of SARS COV2 virus.

### Outcomes assessment

COVID-19 infection was monitored by repeated PCR at least fortnightly. This was mandated by national and local occupational policies. Front-line individuals were required to undergo Nasopharyngeal RT qPCR testing for SARS COV 2 fortnightly in an accredited laboratory. All SEHA accredited laboratories performed the PCR tests utilizing approved kits and following the national testing standards^8^. Clinical classification of severity was assessed and documented by clinical officers as per the WHO COVID-19 clinical management guidance which classifies COVID-19 disease as mild, moderate, severe and critical. Mild included symptomatic patients without evidence of pneumonia, moderate includes pneumonia, severe disease includes severe pneumonia and critical disease includes Acute respiratory distress syndrome (ARDS)^9^.

### Serological testing

Baseline serological testing: At baseline, the anti-S- antibody titer of each individual was determined utilising the cobas® Elecsys Anti-SARS-CoV-2 S assay (Roche Diagnostics, Mannheim, Germany). Positive tests were re-tested with a quantitative assay.

Follow-Up serological testing: Positive baseline serology and samples taken during follow-up visits were investigated with Anti-SARS-CoV-2 QuantiVac ELISA (IgG) (Euroimmune Medizinische Labordiagnostika, Luebeck, Germany) to establish the IgG anti-S1 antibody titer. The test uses 6 calibrator sera to generate a standard curve, and has a reported threshold of < 8 RU/ml (“negative”), ≥ 8 to < 11 RU/ml (“borderline”) and ≥ 11 RU/ml (“positive”). For the study, borderline results were considered “negative”.

All the data was recorded and extracted from the SEHA electronic patient record (EPR) system (Cerner). The data used for the analysis was extracted from the EPR on the 6th of March 2021 and data quality checks were completed by two separate individuals for accuracy.

### Statistical analyses

Baseline characteristics were summarized using descriptive statistics, including mean and standard deviation (SD) for continuous measures, and frequency tables for categorical variables. We compared categorical variables using the Chi-squared or Fisher’s exact tests, and continuous variables using the unpaired t-test or its non-parametric equivalent (Wilcoxon rank sum test) in case the normality assumption is violated.

The primary endpoint was analysed using Kaplan–Meier survival analysis and was defined as the number of days from baseline (i.e. date of second dose + 14 days) until the date of first PCR-confirmed symptomatic Covid-19. Only individuals at risk at baseline (i.e. with negative PCR) were considered in this survival analysis. Individuals who did not experience any symptomatic Covid-19 infection after baseline were considered as right-censored and those who experienced an infection were considered as events. Survival curves were estimated and plotted using the Kaplan–Meier with Efron approach to account for tied data. We also estimated the absolute risk and the incidence rate of COVID-19 infection per 1000-person days. All statistical analyses were performed using R software version 4.0.4^10^. *P* values < 0.05 were considered statistically significant.

### Ethical considerations

The study was approved by the Ethics Review board -Medical Research, Department of health (DOH), Abu Dhabi, UAE (Ref: DOH/CVDC/2021/103). Informed consent was obtained from all the study participants/ legal guardians. The study was performed in accordance with the relevant guidelines and regulations of the ethics review board.

## Results

**Figure 1:**
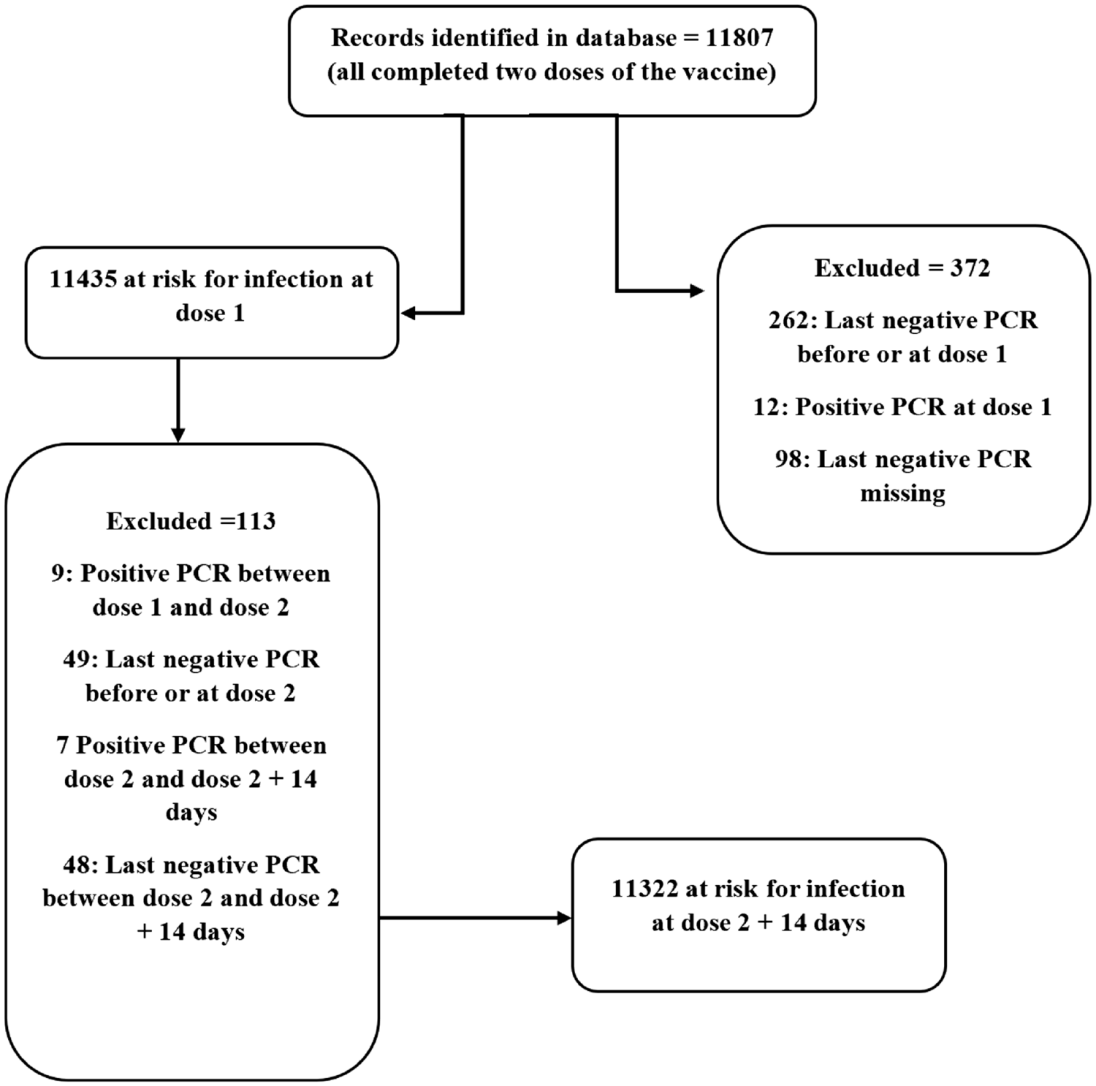
Flow chart explaining inclusion and exclusion for analysis.

A total of 11,807 individuals received the vaccination doses between September 14th 2020, through December 21st 2020, and were followed up consecutively. Among these individuals, 372 were excluded from the current analysis because they were considered not at risk for infection at the date of first dose. A further 113 individuals were also excluded because they were not at risk for infection at the date of second dose plus 14 days. The remaining 11,322 individuals who are PCR negative at 14 days after the date of second dose were considered as at risk of infection and were included in the analysis. (Fig. 1) The median follow-up time for these individuals, calculated from the date of second dose plus 14 days, is 106 days (IQR 98.00–113.00 days).

### Baseline characteristics

Demographic details of the study participants are described in Table 2. Details of all SARS-COV-2 positive individuals are provided in supplementary Table 1. Most participants were male and belonged to the Arab nationality. All individuals fulfilled the Emirates criteria for front line workers as per the national decree^11^. Of the total 11,322 participants, 11,228 (99.17%) remain negative or asymptomatic and 94 (0.83%) became symptomatic with COVID-19 infection. The mean age was 38 ± 10.1 years and less than 10% of the participants had atleast one associated comorbidity.

**Table 2:** Demographic and clinical characteristics of the study individuals.

Characteristics	SARS-COV-2 negative or Asymptomatic (*n*- 11,228) *n* (%)	SARS-COV-2 positive and Symptomatic (*n*- 94) *n* (%)	Total (*n*- 11,322) *n* (%)
Age category			
< 50 years	9716 (86.53%)	84 (89.36%)	9800 (86.56%)
≥ 50 years	1512 (13.47%)	10 (10.64%)	1522 (13.44%)
Gender			
Male	9227 (82.18%)	70 (74.47%)	9297 (82.11%)
Female	2001 (17.82%)	24 (25.53%)	2025 (17.89%)
Nationality			
Arab	6904 (61.49%)	84 (89.36%)	6988 (61.72%)
Asian	3799 (33.84%)	6 (6.38%)	3805 (33.61%)
Other	286 (2.55%)	2 (2.13%)	288 (2.54%)
Missing	239 (2.13%)	2 (2.13%)	241 (2.13%)
Associated comorbidities			
Any comorbidity	1054 (9.39%)	15 (15.96%)	1069 (9.44%)
Diabetic, *n*(%)	442 (3.94%)	9 (9.57%)	451 (3.98%)
Hypertension	617 (5.50%)	9 (9.57%)	626 (5.53%)
Cancer	109 (0.97%)	0 (0.00%)	109 (0.96%)
Pulmonary diseases	20 (0.18%)	0 (0.00%)	20 (0.18%)
Immunosuppression	9 (0.08%)	0 (0.00%)	9 (0.08%)
Transplant	3 (0.03%)	0 (0.00%)	3 (0.03%)
Other comorbidities	183 (1.63%)	3 (3.19%)	186 (1.64%)
Number of comorbidities			
None	10,174 (90.61%)	79 (84.04%)	10,253 (90.56%)
1	717 (6.39%)	11 (11.70%)	728 (6.43%)
≥ 2	337 (3.00%)	4 (4.26%)	341 (3.01%)

### Incidence of COVID-19 infection

A total of 153 individuals had a positive SARS COV 2 PCR after 14 days of the second dose of vaccination (baseline). Of those 153 only 94 had symptomatic infection among whom 92 individuals were diagnosed as having mild COVID-19 disease, while 2 were classified as having moderate disease and required hospital admission for COVID -19 related illness. None of the individuals required intensive care admission.

The estimated incidence rate of COVID-19 infection in the observation period was 0.13 infections per 1000-person days (95% CI 0.11–0.16). The estimated absolute risk of developing a COVID-19 infection was 1.61% (95% CI 1.35%, 1.87%). The risk of suffering from an infection within 90 days after baseline (i.e. second dose + 14 days) was estimated at 1.18% (95% CI 0.97%-1.39%).

**Figure 2:**
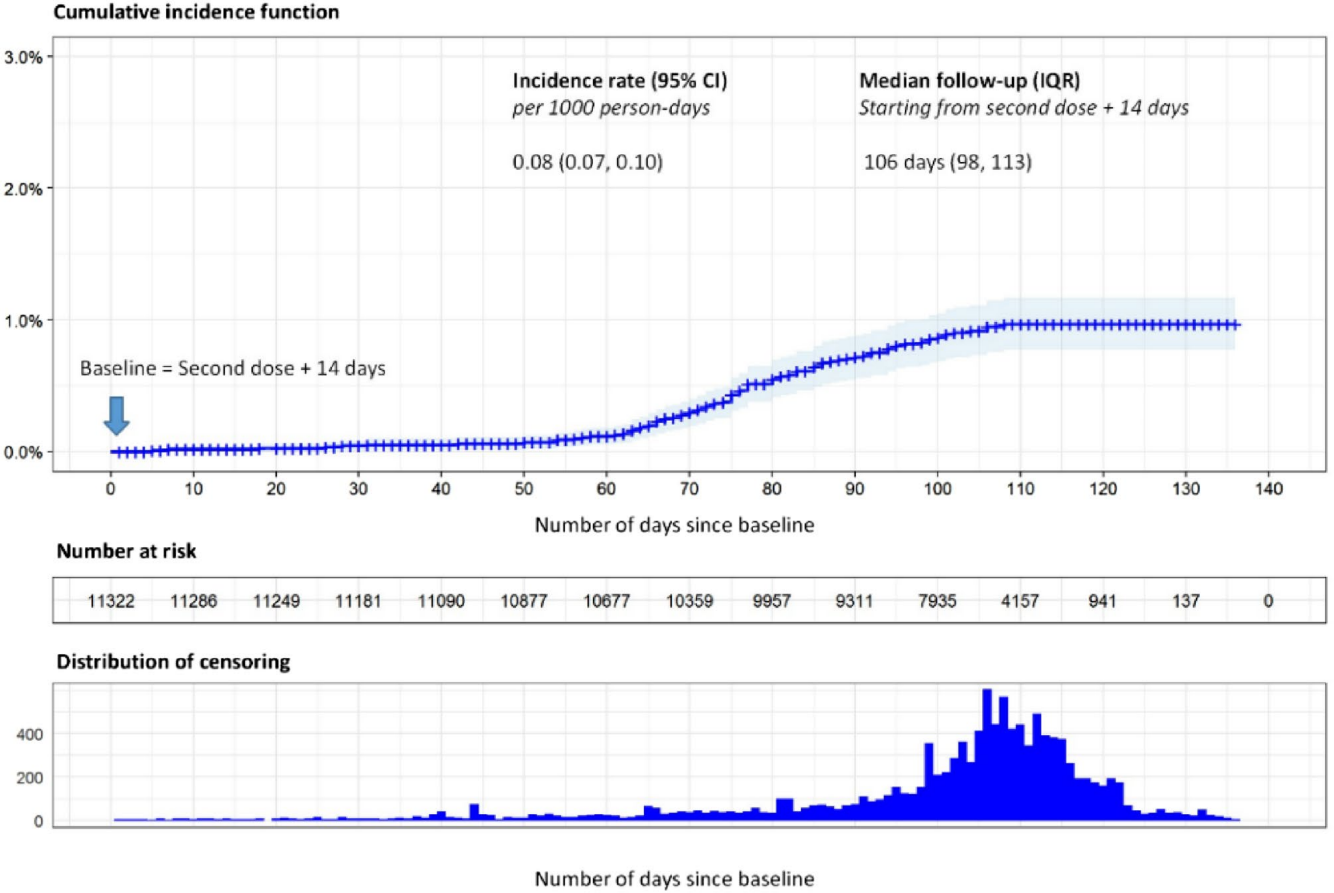
Kaplan–Meier estimates of cumulative incidence rate.

The estimated incidence rate of symptomatic COVID-19 infection during the observation period was 0.082 symptomatic infections per 1000-person days (95% CI 0.06–0.10). The estimated absolute risk of developing a symptomatic COVID-19 infection was 0.97% (95% CI 0.77%, 1.17%). The risk of suffering from a symptomatic COVID-19 infection within 90 days after baseline (i.e. second dose + 14 days) was estimated at 0.72% (95% CI 0.56%–0.88%) (Fig. 2).

### Seroconversion (immunogenicity)

Pre and post vaccination antibody results were available for 10,943 individuals representing 97% of the total participants. 5.9% vaccinated individuals had positive antibodies pre-vaccination. 92.8% of the vaccinated individuals had seroconversion from a negative antibody test to a positive anti body test. About 7.2% of the participants remained seronegative for Anti-SARS-COV-2 QuantiVac ELISA (IgG) following the 2 doses of vaccination (Table 3).

**Table 3:** Seroconversion (Pre and post antibody).

Antibodies pre-vaccination	Antibodies post two doses of vaccination		
	Negative/borderline	Positive	Total
Negative/borderline	740	9556	10,296
Positive	0	647	647
Total	740	10,203	10,943

*p value < 0.0001.

### Safety analysis

**Figure 3:**
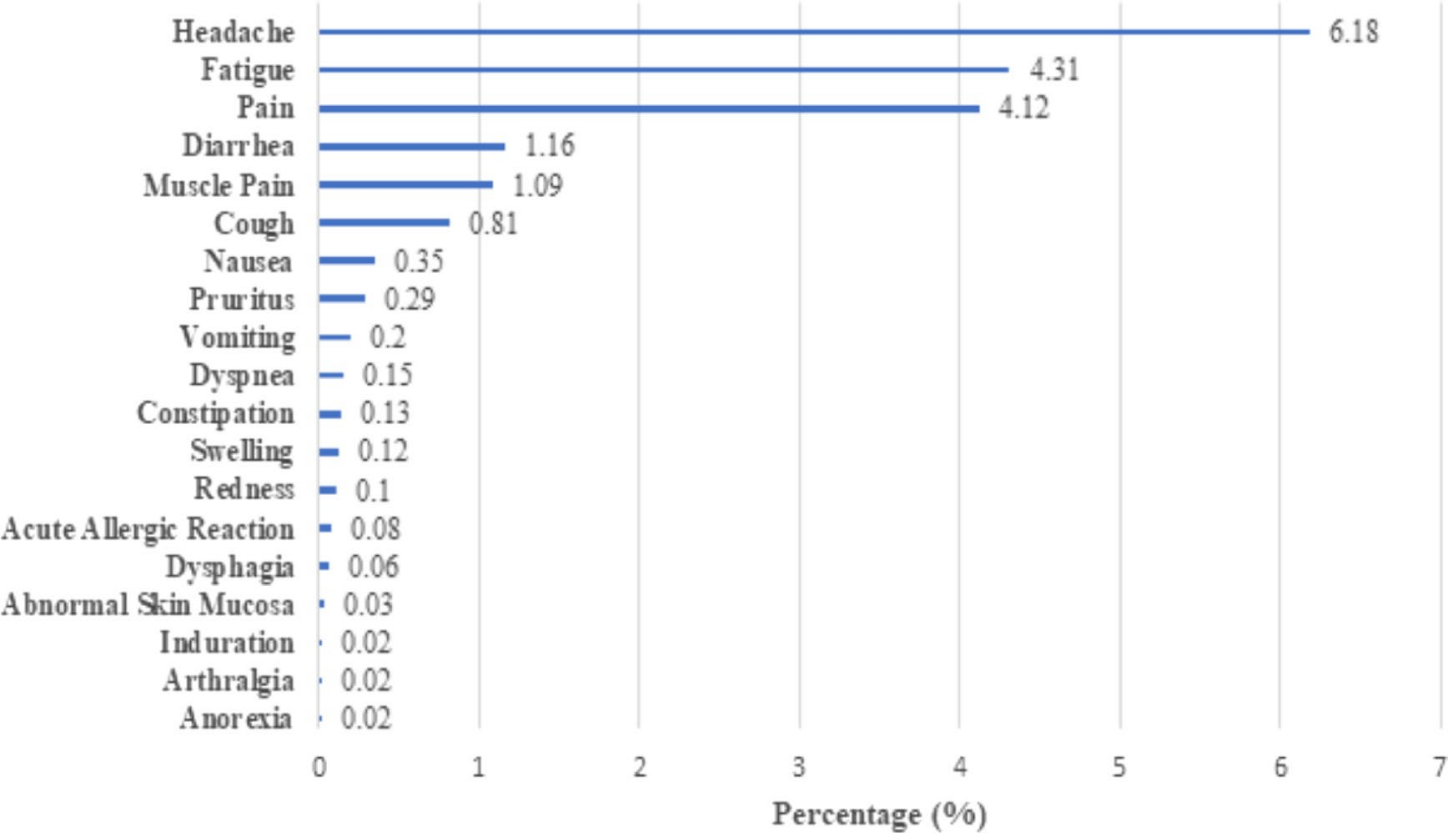
Side effects following vaccination.

Our study surveyed the side effects experienced by individuals who received the vaccination. Out of 11,322 individuals, 1698 (15%) reported having side effects. The most prevalent side effects were headache, fatigue, pain and no severe adverse reactions were reported. (Fig. 3).

## Discussion

Since the pandemic and the rapid development of vaccines, there has been a rapid review of the vaccines available and emergency use authorisation of several candidates by the respective regulatory bodies^12,13^. As such and due to the nature of the current emergency collecting post authorisation data is necessary for further assessment and evaluation of vaccine efficacy and effectiveness^14^.

The WHO published measures of efficacy define a preference for at least 70% efficacy with endpoints being assessed versus disease, severe disease and/or shedding/transmission, with a minimum clear demonstration of efficacy with an ~ 50% point estimate, and an endpoint being assessed versus disease severe disease and or shedding/transmission^15,16^. Assessing the effectiveness of any of the new vaccines is complicated by many factors including, but not limited to, the strains in circulation, the rate of transmission, level of exposure, and individual risk factors; and may not be always reflective of the vaccine efficacy^16^.

Studies show that BNT162b2 vaccine have demonstrated an efficacy of 95% (95% CI- 90.3 to 97.6) in preventing COVID-19 infection in the age group of 16 years and above^17^. Phase III trials results of BBIBP-CORV reported vaccine efficacy against symptomatic infection of 78.1% (95%CI, 64.8%-86.3%)^18^.

In our study we observed an absolute risk of 0.97% of developing symptomatic infection 14 days following the second dose and none of the individuals suffered from severe disease or required admission to intensive care. In a similar study investigating mRNA vaccines among health care workers at the University of California Los Angeles and the University California San Diego, an absolute risk of infection of 1.19% and 0.97% respectively were observed^19^. In an interim estimate of vaccine effectiveness of mRNA vaccines among frontline workers in eight US locations, 0.04 infections per 1000 person-days were reported among individuals who received two doses of vaccines. Those who received one vaccine had an incidence rate of infection of 0.19 per 1000 person-days. Unvaccinated individuals experienced 1.38 infections per 1000 person-days^20^.

In Texas, the percentage of fully vaccinated employees who became infected at the University of Texas Southwestern Center (UTSW) was found to be 0.05% but to the best of our knowledge no incidence rate of infection was reported in their study^21^. In a prospective study among vaccinated staff working in hospitals in the United Kingdom who received the BNT162b2 mRNA or ChADOx1 NCOV-19 adenoviral vector vaccine, the incidence rate of 0.8 per 1000 persons days of follow-up was observed^22^. As per previous studies, our incidence rate is within the range of observed rates for vaccinated frontline workers. Heterogeneity between different reported incidence rates and absolute risks (including clinical trials finding) may be explained by the difference in incidence and prevalence of infections during the study periods and mitigation interventions by different authorities such as lockdowns and compulsory mask usage. For example, during our study period the R_0_ in the UAE increased from 0.95 to a peak of 1.35^23^. In addition, the frequencies of repeated PCR are different across studies, bearing in mind the increase of false positive detection rate in area of low prevalence. This difference in frequency of testing may lead to under or over reporting of infections. In addition, frontline workers are considered at an increased risk of exposure compared to the general population used in clinical trials. The observed differences in the estimated incidence rates across studies maybe also be influenced by the methodology used in survival analysis. Different demographics may also affect the incidence and transmission rate as has been previously noticed. For instance, a surge of transmission was influenced by younger population 20 to 49 years of age^24^. This applies to the UAE as the median age is 32.6 years.

Of interest Pennington et al.^25^ observed, in a large observational study and on review of risk of clinical severity in non-vaccinated individuals by age and sex amongst adults hospitalised with COVID 19, an admission rate of 13.9% in this age group (18–39 years old) with a third of them requiring admission to intensive care, and an increased likelihood of severe infections in the male gender. Our cohort had an over-representation of this particular group, and none of those vaccinated who tested positive for the SARS COV2 virus had any severe complications requiring ICU admission with only 3 individuals requiring hospital admission, two of whom had moderate symptoms and a third individual required admission for non COVID related reasons. No severe adverse side effects were reported and three commonly  reported side effects were headache, fatigue and pain and were most commonly observed following the first dose.

In this study, we have observed a seroconversion rate of 92.8% which is slightly lower than the seroconversion reported earlier in the phase 1/2 trial results of the BBIBP-CORV vaccine^5^. This difference might be due to different sensitivities of the serological assays and methods used for detecting antibodies and the demographics of the population. The serological assay used in this study were developed later in the market and had sensitivity and specificity of more than 90% and 99% respectively, while in the earlier phase I/II trials inhouse developed ELISA and live virus neutralizing antibodies were used. Furthermore, phase I/II trials included only healthy individuals whereas our study had 10% of the population with comorbidities and about 82% of our study population were males who have been observed to have lower antibody response compared to women following inactivated SARS-COV-2 vaccination^26^.

## Limitations

A key limitation of our study is the absence of a control arm defined by non-vaccinated individuals. During the study period, the positivity percentage (i.e. the percent of positive tests from all tests performed in the UAE) ranged between 0.62 and 2.67%, with the peak occurring in January. This observed percentage could be used as a proxy for the prevalence of positive cases in the country, but cannot be compared to the incidence rate or the absolute risk of infection calculated in this study. During the study period, other events may have contributed to the observed infection rate such as improved infection control practices by the individuals, reduced incidence of the infection circulating in the community, and national policies restricting exposure. Further work on this cohort is ongoing with the inclusion of matched controls. The mean age of the cohort was 38, with 81% of those vaccinated being males, and as such individuals at an increased risk of complications (severe disease) are not represented, despite this the absence of any severe disease in this group indicates a possible beneficial effect of the vaccine. The median follow-up period was around 4 months post the second dose. While this was sufficient to derive consistent estimates, longer follow up studies are needed to strengthen this evidence.

## Conclusion

This prospective cohort study is the first to confirm the safety and immunogenicity of a two-dose regimen, 21 days apart of the BBIBP-CORV inactivated vaccine outside of a clinical trial. Based on our findings we conclude that vaccinated individuals at high risk of infection due to their working conditions are most likely to remain PCR negative for SARS COV 2 following the second dose of the vaccination. Additionally, our findings support the relative safety of the BBIBP-CORV as a vaccine candidate and show that none of the vaccinated participant in this cohort suffered from a severe COVID-19 illness.
